# Editorial: Dopaminergic control of experience encoding, memory and cognition

**DOI:** 10.3389/fnbeh.2023.1230576

**Published:** 2023-07-11

**Authors:** Josué Haubrich, Hardy Hagena, Marian Tsanov, Denise Manahan-Vaughan

**Affiliations:** ^1^Medical Faculty, Department of Neurophysiology, Ruhr University Bochum, Bochum, Germany; ^2^Beaumont Hospital, Dublin, Ireland

**Keywords:** dopamine receptors, learning and memory, synaptic plasticity, hippocampus, prefrontal cortex, amygdala

Dopamine plays a central and multifaceted role in several cognitive functions. It is synthesized and released by dopaminergic neurons that originate from different brain regions, mainly the ventral tegmental area (VTA) and the substantia nigra, and innervate several brain areas, including the nucleus accumbens, amygdala, medial prefrontal cortex, striatum, and hippocampus (Höglinger et al., 2014; Beier et al., 2015). Dopamine exerts its effects through two major types of receptors: D1/D5 and D2-like receptors, which activate distinct signaling pathways and modulate synaptic plasticity and neuronal activity. The importance of dopamine in brain functioning is underscored by its well-known involvement in several neurological and psychiatric disorders, such as Parkinson's disease, schizophrenia, depression, and addiction (Klein et al., 2019). Moreover, dopamine-based treatments often disrupt the balance of the system and cause unwanted side effects in patients. Recent research has shed light on the broad involvement of dopamine in the normal functioning of the brain. Dopamine plays an intricate role in the encoding of experiences, a process that intersects with learning and memory, decision-making, novelty processing, motivation, sleep, and attention. This Research Topic addresses these issues with a focus on learning and memory and presents relevant findings that advance our understanding of the role of dopamine in the brain.

How the different dopamine receptors affect brain functions is not well understood and was one of the questions addressed in the current Research Topic. Matzel and Sauce discuss that the balance of D1/5R and D2R with regard to their expression density, activity state, and availability may play a key role in attention and working memory, and in variations in intelligence. In line with this idea, Hagena et al. demonstrate that two mouse strains (CaOlaHsd and C57Bl6) have different patterns of D1/5R expression in the hippocampus and found that such altered balance results in changes in dopaminergic-dependent synaptic plasticity. Thus, differences in D1/D5R expression may explain, in part, strain-dependent variations in synaptic plasticity in the murine hippocampus. The complex relationship between reward, motivation, dopamine, and learning prediction error coding remains a challenging question. The contribution by de Oliveira Alves et al. examines the influence of sex and the estrous cycle on the effects of a D2R-antagonist on contextual fear conditioning in rodents and reports that the involvement of D2R during contextual conditioned freezing response shows specificity in female rats. They show that a D2R-antagonist reduces fear expression only in female rats, especially during the proestrus/estrus phases. These articles show that variations in dopaminergic receptor expression across strains, sexes, and individuals have an important— and perhaps underappreciated—role in synaptic plasticity and cognitive functions.

Another research challenge comprises understanding how dopaminergic neuromodulation in different brain circuits affects cognition. Aberg and Paz report that average reward rates enable motivational transfer across independent reinforcement learning tasks. The mini-review by Zafiri and Duvarci explores the role of various dopaminergic circuits in associative aversive learning, focusing on the amygdala and medial prefrontal cortex circuitry. They highlight how understanding neural circuitry underlying dopamine-dependent aversive learning and memory has high clinical relevance. The review by Sheynikhovich et al. examines dopaminergic modulation of the prefrontal cortex in relation to synaptic plasticities and executive functions, such as spatial strategy selection, memory formation and storage, and extinction learning. The authors show how rodent mPFC is directly involved in long-term memory storage and that memory formation is highly dependent on dopaminergic innervation. In the contribution by Park, the circuit-specific role of D1R on learning and behavioral flexibility was investigated. The author reports that environmental novelty evokes learning-associated plasticity in the vHPC-mPFC and VTA-vHPC circuits, which is dependent on the activation of D1R in the ventral hippocampus. They postulate that this process helps to flexibly overcome previously established spatial bias.

This Research Topic also explored the role of dopamine in human health and disease. Aberg and Paz describe how the average reward rate (ARR) affects subsequent motivation and performance in a novel human reinforcement learning paradigm. They found that a low reward probability impairs performance and that this effect can transfer across independent tasks. The study by Isotalus et al. asked the important question of whether L-DOPA, a dopamine precursor used to treat Parkinson's disease, affects sleep and memory. They show that the timing of L-DOPA application relative to sleep augments non-REM sleep and has the potential to disrupt long-term memory in patients with Parkinson's Disease.

Open questions about the role of dopamine in experience encoding, memory, and cognition can be grouped into two main research domains: fundamental neuroscience and clinical applications. Clinical applications could target three main groups of disorders in the following ways: (1) movement disorders within the nigro-striatal system and dopamine-based therapies require further scrutiny concerning their efficacy in diseases such as Parkinson's disease, dystonia, and Huntington's disease. (2) Memory impairments in neurodegenerative disorders require more clinical trials to elucidate how the pharmacological regulation of dopaminergic neurotransmission may aid patients suffering from neurodegenerative dementias. (3) Psychiatric disorders such as depression, anxiety, panic disorder, and PTSD would strongly benefit from more clinical trials exploring the dopaminergic control of memory consolidation for aversive memories. In fundamental neuroscience, dopamine could be subjected to further scrutiny at three levels: (1) on a cellular level, one should further address how D1/5R and D2R affect different types of neurons and information processing; (2) on a network level, one should further explore how dopamine regulates the functional connectivity of large neuronal populations, particularly in structures that are known to mediate behavior, such as the extended hippocampal system, prefrontal cortex, amygdala, and striatum; (3) on a behavioral level, how theoretical models of reinforcement learning relate to their biological substrates, and how dopamine shapes these to modulate cognitive function, should be addressed in more detail.

In conclusion, this collection of articles highlights the crucial role of dopaminergic modulation in different aspects of synaptic plasticity and cognition. The studies reveal that variations in the expression of dopaminergic receptor subtypes across rodent strains, sexes, and individuals can have profound effects on brain function related to learning and memory. The articles also shed light on the role of dopamine in human motivation, sleep, and brain dysfunction, pointing toward potential therapeutic targets. Overall, these findings open up exciting avenues for future research in the field of dopamine and cognitive neuroscience.

## Author contributions

All authors listed have made a substantial, direct, and intellectual contribution to the work and approved it for publication.
